# The Neurotropic Black Yeast *Exophiala dermatitidis* Induces Neurocytotoxicity in Neuroblastoma Cells and Progressive Cell Death

**DOI:** 10.3390/cells9040963

**Published:** 2020-04-14

**Authors:** Teja Lavrin, Tilen Konte, Rok Kostanjšek, Simona Sitar, Kristina Sepčič, Sonja Prpar Mihevc, Ema Žagar, Vera Župunski, Metka Lenassi, Boris Rogelj, Nina Gunde Cimerman

**Affiliations:** 1 Biotechnical Faculty, University of Ljubljana, 1000 Ljubljana, Slovenia; rok.kostanjsek@bf.uni-lj.si (R.K.); kristina.sepcic@bf.uni-lj.si (K.S.); 2 Institute of Biochemistry, Faculty of Medicine, University of Ljubljana, 1000 Ljubljana, Slovenia; tilen.konte@gmail.com (T.K.); metka.lenassi@mf.uni-lj.si (M.L.); 3 Laboratory for Polymer Chemistry and Technology, National Institute of Chemistry, 1000 Ljubljana, Slovenia; simona.sitar84@gmail.com (S.S.); ema.zagar@ki.si (E.Ž.); 4 Veterinary Faculty, University of Ljubljana, 1000 Ljubljana, Slovenia; sonja.prparmihevc@vf.uni-lj.si; 5 Faculty of Chemistry and Chemical Technology, University of Ljubljana, 1000 Ljubljana, Slovenia; vera.zupunski@fkkt.uni-lj.si (V.Ž.); boris.rogelj@ijs.si (B.R.); 6 Department of Biotechnology, Jožef Stefan Institute, 1000 Ljubljana, Slovenia; 7 Biomedical Research Institute, 1000 Ljubljana, Slovenia

**Keywords:** neurotropic fungi, black yeasts, neurotropism, hydrocarbons, extracellular vesicles, Alzheimer’s disease, human neuroblastoma cells, SH-SY5Y, *Exophiala dermatitidis*

## Abstract

The neurotropic and extremophilic black yeast *Exophiala dermatitidis* (*Herpotrichellaceae*) inhabits diverse indoor environments, in particular bathrooms, steam baths, and dishwashers. Here, we show that the selected strain, EXF-10123, is polymorphic, can grow at 37 °C, is able to assimilate aromatic hydrocarbons (toluene, mineral oil, n-hexadecane), and shows abundant growth with selected neurotransmitters (acetylcholine, gamma-aminobutyric acid, glycine, glutamate, and dopamine) as sole carbon sources. We have for the first time demonstrated the effect of *E. dermatitidis* on neuroblastoma cell model SH-SY5Y. Aqueous and organic extracts of *E. dermatitidis* biomass reduced SH-SY5Y viability by 51% and 37%, respectively. Melanized extracellular vesicles (EVs) prepared from this strain reduced viability of the SH-SY5Y to 21%, while non-melanized EVs were considerably less neurotoxic (79% viability). We also demonstrated direct interactions of *E. dermatitidis* with SH-SY5Y by scanning electron and confocal fluorescence microscopy. The observed invasion and penetration of neuroblastoma cells by *E. dermatitidis* hyphae presumably causes the degradation of most neuroblastoma cells in only three days. This may represent a so far unknown indirect or direct cause for the development of some neurodegenerative diseases such as Alzheimer’s.

## 1. Introduction

Polyphyletic black yeasts, including the genus *Exophiala*, are melanised yeast-like fungi that populate extreme environments dominated by high or low temperatures, high salinity, aridity, low water activity, high UV radiation, fluctuating pH, and oligotrophicity [1,2,3,4,5,6,7,8]. They have a distinct extremophilic ecotype, characterized by thick, melanised cell walls, as well as slow, polymorphic growth including hyphae, yeast cells, meristematic clumps, and endoconidiation. Many black yeasts, in particular those within *Chaetothyriales*, fam. *Herpotrichiellaceae*, use the above exaptations and their unusual ability to grow at 37 °C to invade the human body, another extreme environment for fungi [3].

*Exophiala dermatitidis* is the most clinically important and thermotolerant species of the genus *Exophiala*. It can cause various medical conditions, from cutaneous and subcutaneous infections, to systemic, gastrointestinal, pulmonary, and neurotropic infections [9]. It has been isolated from the ears, sinuses, lungs, mucus of cystic fibrosis patients, blood and different catheters, and most importantly brain infections [3,10,11,12,13,14,15,16,17,18,19,20,21]. Occasionally, it can cause mildly invasive systemic infections that are associated with significant morbidity and mortality. Typical infections are seen in immunocompromised hosts such as transplant recipients, oncology, and pediatric patients, where it manifests itself as a subcutaneous disease and rarely as deep mycoses [9,22,23,24,25,26]. Particularly in East Asia, *E. dermatitidis* was detected in disseminated and neurotropic infections with high mortality [11,27]. Note, however, that despite infections occurring in apparently healthy humans, some authors concluded that *E. dermatitidis* and related species are opportunists rather than pathogens [28,29,30].

Its pathogenicity is probably owing to various virulence factors, including melanin pigmentation, thermotolerance, and polymorphism [9]. Melanin accumulates in the cell wall of *E. dermatitidis*. This has a protective effect against harmful substances and oxidative stress from the environment or the host cell [31]. *Exophiala* species synthesize melanin endogenously from acetate via the pentaketide pathway, leading to 1,8-dihydroxynaphthalene melanin. This pathway can be constrained under controlled growth conditions by addition of inhibitors, thus allowing the study of melanin’s relations to morphology, physiology, and pathogenicity [32,33,34].

The polymorphic character and melanin pigmentation of *E. dermatitidis* enable its colonisation under stress conditions. *Exophiala dermatitidis* has adapted to human-made indoor environments, such as steam baths, saunas, public baths [35,36], drainpipes, and drinking water. It is most frequently (and globally) present in domestic dishwashers [37,38,39], where internal rubber seals and plastic parts can harbour up to 10^6^ colony forming units/cm^2^ [39]. As people spend more time indoors and the number of immunocompromised people is rapidly increasing, the establishment of *E. dermatitidis* in domestic environments represents an important risk factor for human health [40,41].

Fungi normally populate parts of the human body, for example, skin, mucus of respiratory tract, oral cavity, and mucus of the digestive tract [28]. They most frequently invade through the respiratory tract by inhalation of spores or mycelium, but also enterically via the gastrointestinal tract or via traumatic injuries (e.g., accidents, surgery, interventions) [3]. After the initial infections, the fungi can spread via the haematogenous route. Fungal infections of the central nervous system occur either indirectly via lungs or paranasal sinuses, for example, after near-drowning episodes [42,43,44,45], via ocular orbits, and mastoid region of the temporal bone or retropharyngeal area, or directly as a consequence of trauma, invasive treatments, or brain surgery [41]. The potential mechanism of invasion of *E. dermatitidis* and other neurotropic fungi via the peripheral nervous system has not yet been described, nor have the mechanisms contributing to the spread of fungal infections to the brain.

*Exophiala dermatitidis* is rare in nature, but its occurrence increases in environments contaminated with cyclic or non-cyclic aromatic hydrocarbons [5,6,8] such as creosote-treated or oil-contaminated railway beams [46,47]. It can also be isolated from the cuticle of ants and ant hills [48,49], but most frequently on artificial rubber seals of dishwashers [37]. The neurotropic potential of black yeasts within *Chaetothyriales*, including *E. dermatitidis*, has been closely associated with their ability to assimilate monoaromatic and polyaromatic hydrocarbons, hypothetically including phenolic and aliphatic metabolic degradation products of catecholamine-like neurotransmitters [6,8,50,51,52]. It is known that disturbed transport or lowered concentrations of neurotransmitters can lead to neurodegenerative diseases, such as Alzheimer’s [6,50,53].

According to recent investigations, systemic mycoses could be either one of the causative agents or an additional risk factor for the development of Alzheimer’s. Fungal macromolecules were detected in the peripheral vascular system and in the cerebrospinal fluid (CSF) from patients with the disease [54,55], and elevated chitinase levels were also detected in the CSF [56]. Fungal yeast cells and hyphal fragments were detected in different parts of the brain, both inside and outside the neurons. In spite of this and other evidence [57,58], there is still ambiguity regarding the etiological role of fungi in Alzheimer’s [57,59].

Fungi have also been associated with other neurological diseases. Recently, an increasing number of opportunistic mycoses of the central nervous system (CNS) has been reported in healthy individuals and, in particular, in patients with sepsis, prolonged ventilation, oncological therapies, organ transplantation, overuse of antibiotics, HIV patients, and others [41,60]. Opportunistic mycoses of CNS are associated with higher morbidity and mortality [61,62] owing to pathogenic fungi such as *Cryptococcus neoformans,* which are able to cross the otherwise prohibitive blood–brain barrier [41,63]. 

Over the last decades, extracellular vesicles (EVs) were identified as potential mediators of intra-cellular and inter-organism communication in all life kingdoms [64,65]. The first evidence of fungal EVs came from the opportunistic human pathogen *Cryptococcus neoformans* [66]. From that time, studies on the roles of EVs in pathogenicity of other fungi increased significantly (reviewed in Bielska and May) [67]. Fungal EVs are heterogeneous populations of lipid-bilayer nanoparticles that harbour cargo molecules important in modulating virulence, host defence, and host immune function, as well as triggering anti-microbial activities [67]. *Cryptococcus neoformans* EVs can cross the blood–brain barrier and accumulate as lesions in the brain, facilitating adhesion and transcytosis [68,69,70,71].

To extend our understanding of *E. dermatitidis* virulence factors associated with its neurotropic character, we investigated various aspects of the effect of *E. dermatitidis* on the human neuroblastoma cell line SH-SY5Y. These include viability assays after adding fungal metabolites or EVs and by direct exposure of neuroblastoma cells to *E. dermatitidis* cells to determine its potential neurocytotoxicity.

## 2. Materials and Methods

### 2.1. Strains and Growth Conditions

*Exophiala dermatitidis* EXF-10123 strain (CBS 525.76; isolation source: human) was obtained from the Culture Collection Ex Infrastructural Centre Mycosmo, part of MRIC UL, Department of Biology, Biotechnical Faculty, University of Ljubljana, Ljubljana, Slovenia. Cultures were maintained on malt extract agar (MEA), incubated at 37 °C, for up to seven days. For the determination of morphological features, the fungus was cultured on MEA and oat meal agar (OA) and incubated for two weeks. For all other experiments, it was cultured on defined yeast nitrogen base (YNB) medium: 17% (w/v) YNB (Formedium, Hunstanton, UK), 0.5% (w/v) ammonium sulphate (Merck Millipore, Darmstadt, Germany), 2.0% (w/v) D-glucose in deionised water, pH 7.0. Fungal cell suspensions for the inoculation of media used for the determination of the assimilation of hydrocarbons and neurotransmitters were prepared by growing *E. dermatitidis* on MEA and preparing a suspension of 10^5^ cells/mL. In addition, *S. cerevisiae* Y00000 (S288c) wild type strain, provided by the Euroscarf collection (Scientific Research and Development GmbH, Oberursel, Germany), was cultured in the same media and at the same conditions as *E. dermatitidis*.

### 2.2. Cell Culture

SH-SY5Y cells (ATCC^®^ CRL-2266) were cultured according to standard protocol [72]. Briefly, cells were cultured in Dulbecco’s modified Eagle’s medium/Nutrient Mixture F-12 (DMEM/F-12 GlutaMAX; Gibco, Thermo Fisher Scientific, Waltham, MA, USA), supplemented with 10% (v/v) FBS and 1x Penicillin-Streptomycin (10,000 U/mL). Cells were grown at 37 °C in a humidified atmosphere with 5% CO_2_. SH-SY5Y cells were confirmed to be Mycoplasma negative using the MycoAlert™ Kit (Lonza, Basel, Switzerland), following the manufacturer’s protocol. For functional tests, the adherent form of SH-SY5Y cells between the 26th and 36th passages was used.

### 2.3. Morphological Observations

A sample of fungal culture grown on MEA and OA was detached by adhesive tape that was placed on the slide and mounted with a drop of Aniline Blue (2% solution in lactic acid; Sigma Aldrich, St. Louis, MO, USA) for cytoplasm staining. Images were captured using a light microscope Olympus BX51 equipped with differential interference contrast (DIC; Olympus, Tokyo, Japan).

### 2.4. Assimilation of Hydrocarbons

The assimilation of hydrocarbons was determined in both liquid medium and solid plates, according to Satow et al. [73] and Prenafeta-Boldú et al. [5] and modified referring to the protocol of Zajc et al. [74]. In the case of liquid media, YNB was supplemented with 20% (v/v) of mineral oil (Sigma Aldrich, USA) and 20% (v/v) of n-hexadecane (Sigma Aldrich, USA) as sole carbon sources. Tubes were inoculated with 100 µL fungal cell suspension (10^5^ cells/mL) and incubated on a rotary shaker (180 rpm) at 25 °C for two weeks. Additionally, plates with YNB solid medium were inoculated with the cell suspension and incubated at room temperature in a desiccator under a 3% toluene atmosphere (v/v) solution in dibutyl phthalate (Sigma Aldrich, USA). The growth was monitored weekly and the size diameter of the colonies compared with the control plates incubated without toluene vapours.

### 2.5. Assimilation of Neurotransmitters

For assessing the fungal assimilation of neurotransmitters, a modified M9 minimal medium was prepared according to Harwood and Cutting [75]. Each M9 medium (pH 7.4) with macronutrients and trace salts was supplemented with one of the five neurotransmitters (0.1 M) (Sigma Aldrich, USA): acetylcholine (ACh), γ-aminobutyric acid (GABA), glycine (Gly), glutamate (Glu), and dopamine (DA). As control, M9 medium without the addition of neurotransmitters was used. All plates were inoculated at three points and incubated at 25 °C for four weeks, while Dopa medium was additionally incubated at 37 °C. Colony growth was monitored weekly.

### 2.6. Biomass Extraction

*E. dermatitidis* culture was grown in liquid malt extract (ME) medium, inoculated with 0.2% (v/v) of suspension prepared from *E. dermatitidis* grown on MEA and incubated at 37 °C on a rotary shaker (180 rpm) for 4 days. The cells were harvested by centrifugation at 10,000× *g*, for 10 min at 25 °C. The supernatant was sterilised by filtration through a membrane with a pore size of 0.45 µm and stored at −20 °C until use. Sedimented fungal biomass was separated into two equal parts and deep frozen prior to extraction.

The extraction of water-soluble compounds from fungal biomass was performed with 0.01 M phosphate buffer supplemented with 0.8% (w/v) NaCl and 0.02% (w/v) KCl, pH 7.4 (Sigma Aldrich, USA). The biomass (5.31 g) was first ground in a mortar under continuous cooling with liquid nitrogen, and then extracted for 24 h with 6 mL of the extraction buffer at 4 °C on a rotary shaker (180 rpm). The pellet was then removed by centrifugation at 15,000× *g*, for 30 min at 4 °C. The aqueous extract was sterilised by filtration through a membrane with a pore size of 0.22 µm, aliquoted, and stored at −20 °C until testing on human cell lines. Dry mass of the extracts was determined by gravimetry.

For the extraction of less polar compounds, *E. dermatitidis* biomass (5.03 g) was resuspended in ethanol (3 mL) and incubated at 37 °C on the rotary shaker (180 rpm) for 24 h. The extract was separated by centrifugation at 15,000× *g* for 30 min at 4 °C. Extraction was repeated twice. The solvent was evaporated by a MiVac DUO concentrator (Genevac, Ipswich, UK), and the dry weight of the extract was determined by gravimetry. The dried extract was stored at −20 °C and resuspended in DMSO (Sigma Aldrich, USA) before testing.

### 2.7. Isolation of Fungal Extracellular Vesicles (EVs)

Overnight, *E. dermatitidis* culture was used to inoculate YNB medium with optical density (OD) adjusted to approximately OD_600_ = 0.2 and incubated at 37 °C on a rotary shaker (180 rpm) until reaching the mid-exponential growth phase (OD_600_ = 0.8–1.0). Cells were harvested by centrifugation at 1000× *g* for 10 min at 30 °C, washed three times with fresh YNB medium, and suspended in 400 mL YNB medium at OD_600_ = 0.2. After 15 h incubation at 37 °C on a rotary shaker (180 rpm), EVs were collected from the media. For the functional studies, *E. dermatitidis* was alternatively grown in YNB media supplemented with the melanin inhibitor tricyclazole (5-methyl-1,2,4-triazolo(3,4,-b)-benzothiazole) (Dow Agro Science LLC, Indianapolis, IN, USA) added to the final concentration of 30 μg/mL in 1% (w/v) ethanol solution. Cells were removed by sequential centrifugation at 4000× *g* for 15 min, after which the supernatant was filtered through a 0.22 μm PES filter (TPP, Trasadingen, 7Switzerland) to remove larger particles. The filtrate was concentrated 20-fold in Amicon Stirred Cell concentrator (Merck Millipore, Germany) with 100 kDa PES membrane, and EVs were later pelleted by ultracentrifugation at 100,000× *g* for 1 h 10 min at 4 °C (MLA-50, Optima MAX-XP, Beckman Coulter, Indianapolis, IN, USA). Pelleted EVs were then washed in DPBS (Dulbecco’s phosphate buffered saline; Sigma Aldrich, USA) by ultracentrifugation at the same conditions (TLA-55). Final pellet was resuspended in 80 µL of DPBS and then aliquoted in 40/20/20 uL and stored on ice until use. Functional tests on cell lines were done on the same day.

Alternatively, EVs pelleted by the first ultracentrifugation step were suspended in 400 µL DPBS and centrifuged at 100,000× *g* for 18 h at 4 °C on sucrose gradient composed of 400 µL fractions of 20%, 24%, 28%, 32%, 36%, 40%, 44%, 48%, 52%, 56%, and 60% sucrose (w/v), (Sigma Aldrich, USA) in 0.01 M DPBS. Fractions (400 µL) collected from the top of the gradient were precipitated with trichloroacetic acid/Na-deoxycholate (TCA-DOC), (Sigma Aldrich, USA), as described previously [76] and below, and frozen at −20 °C until further use.

### 2.8. Characterisation of Extracellular Vesicles

Protein extraction and quantification. EVs proteins were extracted by TCA-DOC precipitation with the addition of 100 µL of both 70% (w/v) TCA and 0.15% (w/v) DOC, to fractions diluted to 1 mL in distilled water. The samples were centrifuged at 16,000× *g* for 15 min at room temperature, after which the pellet was washed with ice cold acetone to remove excess of TCA, and later resuspended in 15 µL of radioimmunoprecipitation assay (RIPA) buffer composed of 0.1% (w/v) SDS (Sodium dodecyl sulfate; Sigma Aldrich, USA), 0.5% (w/v) Na-deoxycholate (Sigma Aldrich), 1.0% (v/v) NP-40 (IGEPAL CA-630; Sigma Aldrich), and 0.1% (v/v) protease inhibitor (Thermo Fisher Scientific, USA) in 0.01 M phosphate buffered saline. The total amount of exosomal proteins (μg), isolated from 400 mL of *E. dermatitidis* culture, was determined by the Pierce BCA protein Assay Kit (Thermo Fisher Scientific, USA) following the manufacturer’s instructions.

Melanin quantification. Melanin content was determined by measuring absorbance at 400 nm (A_400_) in 96-well plates by Synergy2 reader (BioTek, Winooski, VT, USA), as published by Forest and Simon (1998) [77].

Asymmetrical-flow field-flow fractionation coupled to a multidetection system (AF4/UV-MALS). AF4 was performed at room temperature on an Eclipse 3+ system (Wyatt Technology Europe, Dernbach, Germany) consisting of an isocratic pump, an online vacuum degasser, and an autosampler (Agilent Technologies 1260 series; Agilent Technologies, Santa Clara, CA, USA). EV samples were separated in a channel with a trapezoidal-shaped spacer with a thickness of 350 μm, a tip-to-tip length of 152 mm, and an initial channel breadth of 21 mm that decreased to a final 3 mm. A 10 kDa regenerated cellulose membrane was utilized as the accumulation wall. The fractionated particles were detected with a UV detector operating at a wavelength of 280 nm (Agilent Technologies, USA), and a multi-angle light-scattering (MALS) detector (DAWN HELEOS, Wyatt Technology, Santa Barbara, CA, USA) equipped with a 658 nm GaAs laser diode. A 90° scattering angle was calibrated with toluene, while other detectors were normalized with bovine serum albumin protein as an isotropic scattered standard. PBS (phosphate buffered saline, pH 7.4) as a running eluent, composed of 137 mM NaCl, 2.68 mM KCl, 10.14 mM Na_2_HPO_4_, and 1.84 mM KH_2_PO_4_, was supplemented with 0.02% w/v sodium azide (NaN_3_) as a bactericide, and filtered through a Nylon-66 membrane with a pore size of 0.45 μm (Supelco Analytical, Sigma Aldrich, USA). Between the HPLC pump and the AF4 channel, an additional filter with a pore size of 0.1 μm was placed (PEEK Inline Filter Holder).

Samples were injected in focus mode at 0.2 mL/min over 3 min. After injection, the samples were focused for additional 2 min. The elution step was performed with two linear cross-flow gradients: cross-flow decreasing from 3.0 to 0.25 mL/min within 5 min and then cross-flow decreasing from 0.25 to 0.09 mL/min within 60 min. The 0.09 mL/min is the lowest cross-flow limit of the instrument. Once this limit is reached, the cross-flow is turned off. The detector flow rate was 1 mL/min. The last step was washing the channel for 10 min in elution mode without using any cross-flow.

ASTRA 5.3.4.20 software from Wyatt Technology (Santa Barbara, USA) was utilized to analyse the acquired data. The size of the exosomes was expressed by two different radii, that is, the root mean square radius (*R*_g_) and the geometric radius (*R*_geom_), which were obtained from the MALS detector without the need for the solute concentration and the sample refractive index increment. The *R*_g_ radii of the fractionated exosomes were calculated using the data from 15 angles from the MALS detector, applying Debye 2nd order model. The *R*_geom_ and the number of exosomes per mL (number density) were determined by the Astra Particle template assuming a spherical exosome shape, and the Astra Number density template using a exosome refractive index of 1.39, respectively [78].

Transmission electron microscopy (TEM). The exosome sample was visualized by TEM using the negative staining method. Exosome suspension (0.12 μg/μL) was applied on Formvar-coated and carbon-stabilized copper grids and contrasted with 1% (w/v) water solution of uranyl acetate. Samples were examined with CM100 (Philips, Amsterdam, The Netherlands) transmission electron microscope, operating at 80 kV. Images were recorded with Orius 200 camera (Gatan) using DigitalMicrograph software v. 2.32 (Gatan Inc., Pleasanton, CA, USA).

### 2.9. Effect of Volatile Organic Compounds (VOCs) of E. dermatitidis on SH-SY5Y Cells

Briefly, 6 × 10^4^ SH-SY5Y cells/cm^2^ were seeded into two middle wells of a 12-well microplate and incubated for 24 h, followed by filling the outer wells with 10^7^/mL of *E. dermatitids* cultured for 1, 2, or 8 days (separate microplate for each incubation time) and incubated for 48 h in cell incubator. To provide sterile conditions with exposure to volatile metabolites, but preventing spreading fungal cells, smoking rolling paper (Ziggi, Ljubljana, Slovenia) was attached to the wells filled with fungal suspensions. After incubation of SH-SY5Y cells at these conditions, viability was visually detected by staining with trypan blue solution (0.4%; Thermo Fisher Scientific, USA) according to Strober [79]. As a positive control, 96% (v/v) ethanol was filled into wells instead of *E. dermatitidis* suspension.

### 2.10. Neutral Red Uptake Assay

Function of *E. dermatitidis* extracts. Neutral red uptake assay was performed to determine cytotoxicity of fungal metabolites (e.g., extracts or EVs) on the human neuroblastoma cell line. The assay was performed using a modified protocol of Repetto et al. [80]. Briefly, 6 × 10^4^ SH-SY5Y cells/cm^2^ were seeded into 96-well microplates and incubated for 24 h to allow for cell attachment, followed by a 48 h treatment with fungal extracts or EVs suspensions prepared in fully supplemented cell culture medium. Final concentrations of extracts were 0.4, 0.9, 1.3, 1.8, 2.6, 3.7, 5.6, and 7.4 mg/mL for aqueous and 0.1, 0.2, 0.5, 0.9, 1.4, 1.8, 2.7, and 3.7 mg/mL for organic extract. The pH of all extracts was adjusted to 7.4 before performing the assay. The final amount of EVs was determined as a concentration of particles per mL, and the EVs were added in the final concentrations of 0.0, 2.3, 4.8, 9.2, and 1.4 (× 10^9^) EVs/mL for melanised and 0.0, 1.0, 2.0, and 3.0 (× 10^9^) EVs/mL for non-melanised EVs calculated from final concentrations in percentage (0%, 8.3%, 17%, 33%, and 50% (v/v)). As a positive control for cytotoxicity, 0.5 mM H_2_O_2_ was used. After treatment, 0.04 mg/mL neutral red dye (Sigma Aldrich, USA) was added to each well and cells were incubated for 2 h, allowing the dye to become trapped inside the lysosomes. Cells were rinsed with PBS, followed by releasing the internalized dye by a prepared solvent: 50% (v/v) ethanol, 1% (v/v) acetic acid, in deionized water. The absorbance of released neutral red dye was measured spectrophotometrically by a microplate reader (Thermo Fisher Scientific, USA) at a wavelength of 550 nm. All cytotoxicity experiments were performed at least twice as independent experimental repeats and in three to five technical repeats.

Function of *E. dermatitidis* conditioned medium. The supernatant of harvested cells for biomass production was used as a conditioned fungal medium (unconcentrated). To assess the function of SH-SY5Y cells after conditioned fungal medium was added, neutral red uptake (NRU) assay was performed using a procedure according to Repetto et al. [80]. Final concentrations for the experiment with conditioned medium (in percentages) were 1%, 2%, 3%, 4%, 6%, 8.3%, 12.7%, and 16.7% (v/v), as well 4% (v/v) ME medium as a control.

Statistical Analysis. The data from all cytotoxicity experiments were expressed as the arithmetic mean ± standard deviation (SD) and were statistically analysed by Kruskal–Wallis analysis. A *p*-value lower than 0.05 was considered statistically significant. All statistical analyses were performed using GraphPad Prism software (GraphPad Software, San Diego, CA, USA).

### 2.11. Scanning Electron Microscopy (SEM)

For scanning electron microscopy (SEM), SH-SY5Y cells were cultured at the seeding density of 3.2 × 10^4^/cm^2^ on coverslips (ø 10 mm), coated with 0.1% (w/v) poly-L-lysine (Sigma Aldrich, USA) placed into 24-well microplates, and incubated for 72 h until they reached approximately 70% confluency. Cell suspension of *E. dermatitidis* was prepared in fully supplemented cell culture medium DMEM/F-12 GlutaMAX. SH-SY5Y cells on coverslips were supplemented with fresh cell culture media and fungal cells at the final concentration of 5.4 × 10^4^/cm^2^ and incubated for one to four days. Incubations were performed in two technical replicates and three independent experimental repeats. After each incubation, interval coverslips with cells were fixed in 1.0% (v/v) glutaraldehyde and 0.4% (v/v) formaldehyde in 0.1 M sodium phosphate buffer (pH 7.2) at 4 °C overnight. After washing in 0.1 M phosphate buffer, the samples were postfixed in 1% aqueous solution of OsO_4_ (SPI-CHEM, West Chester, PA, USA). Postfixed samples were dehydrated in an ethanol series of ascending concentrations (30%, 50%, 70%, 90%, and 100%). Final ethanol concentration was gradually replaced by hexamethyldisilazane (Merck, Germany) and allowed to air-dry overnight. Coverslips with dried samples were attached to the metal holders with silver paint (SPI CHEM, USA), coated with platinum on SCD 050 sputter coater (BAL-TEC, Untersiemau, Germany), and observed with a JEOL JSM-7500F (JEOL Ltd., Tokyo, Japan) filed-emission scanning microscope.

### 2.12. Immunofluorescence

The coverslips with SH-SY5Y cells and *E. dermatitidis* cells were incubated for 24 or 48 h in duplicates. The cells were fixed in 4.0% (v/v) formaldehyde in 0.01 M phosphate buffered saline (pH 7.4) for 15 min, followed by washes with 0.01 M phosphate buffered saline (pH 7.4). Blocking and permeabilization was performed using 5.0% (v/v) FBS with 0.1% (v/v) Triton X-100 in 0.01 M phosphate buffered saline for 30 min at room temperature. The cells were incubated for 1.5 h at room temperature with mouse monoclonal anti-GAPDH antibody (ZG003; 1:1000; Invitrogen, Thermo Fisher Scientific, USA). After washes, the cells were incubated for 1 h at room temperature with anti-mouse Alexa Fluor^®^ 555 IgG H&L (1:1000; Cell Signaling Technology, Danvers, MA, USA) in the dark. To stain yeast cells, the samples were incubated with Calcofluor^®^ White M2R (Thermo Fisher Scientific, USA) diluted (1:200) in wash buffer composed of 2.0% (w/v) D-glucose in 10 mM HEPES buffer for 30 min at 30 °C. After final rinsing, the coverslips were mounted with ProLong Gold Antifade reagent with or without DAPI (Molecular Probes, Thermo Fisher Scientific, USA). Fluorescent images were captured using a confocal laser scanning microscope Leica TCS SP8 with LAS X software (Leica Microsystems GmbH, Wetzlar, Germany). Each experiment (in duplicates) was repeated three times.

## 3. Results

### 3.1. Exophiala dermatitidis Increases Melanisation and Yeast-like Growth at Human Body Temperature

**Figure 1:**
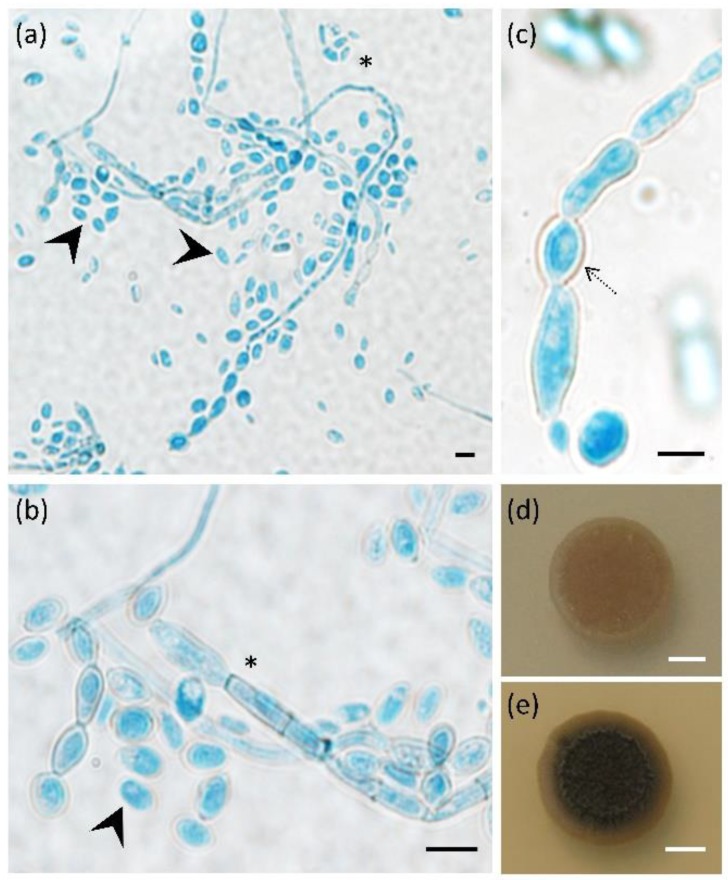
Morphological characteristics of *Exophiala dermatitidis* (EXF-10123). (**a**) Light microscope images (differential interference contrast, DIC) of Aniline Blue stained fungus grown on two different morphological media, (**a**,**b**) oatmeal agar (OA) and (**c**) malt extract agar (MEA); images show three different fungal growth forms, budding cells, (black arrow), pseudo-hyphae (black dotted arrow), and septate hyphae (asterisk). (**d**,**e**) *E. dermatitidis* is capable of growth at 37 °C. Culture on MEA plates incubated at (**d**) 25 °C and (**e**) 37 °C. Scale bar: 5 µm (black), 5 mm (white).

The main morphological characteristics of *E. dermatitidis* strain EXF-10123 (CBS 525.76) were analysed on MEA and OA media, both at room temperature and at 37 °C (Figure 1). All three typical *E. dermatitidis* growth forms were present on both media: conidia, pseudo-hyphae, and hyphae. EXF-10123 grew more vigorously on MEA, while colonies were less melanised and more superficial than on OA. MEA was selected for further tests. In comparison with growth at room temperature, EXF-10123 at 37 °C showed a much higher degree of colony melanisation and abundant excretion of dark pigments. At 37 °C, yeast cells prevailed, while at room temperature, the filamentous form prevailed.

### 3.2. Exophiala dermatitidis Assimilates Selected Hydrocarbons and Neurotransmitters as Sole Carbon Sources

**Figure 2:**
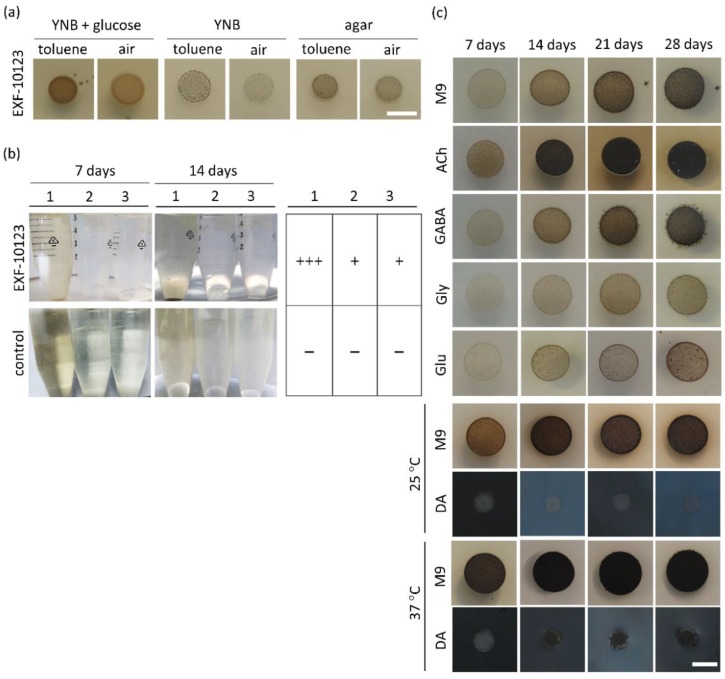
*Exophiala dermatitidis* assimilated selected cyclic aromatic hydrocarbons and neurotransmitters as sole carbon sources. (**a**) Growth of *E. dermatitidis* was assessed on solid yeast nitrogen base (YNB) medium, with and without glucose and ultra-pure agar plates without carbon source added. Plates were incubated for seven days under toluene vapours and compared with a control (without toluene vapours). (**b**) *E. dermatitidis* growth was assessed as well in liquid YNB media with different carbon sources: glucose (1), mineral oil (2), and n-hexadecane (3); incubated for 7 and 14 days; and compared to control (without fungus). Growth rate was qualitatively estimated with a tabular overview. (**c**) Four-week monitored experiment for determining growth and morphological characteristics of *E. dermatitidis* on solid M9 medium supplemented with various neurotransmitters, ACh, GABA, Gly, Glu, and DA, as a sole carbon source. Legend: 1—YNB with glucose, 2—YNB with 20% mineral oil, 3—YNB with n-hexadecane; Ach: acetylcholine, GABA: gamma-aminobutyric acid, Gly: glycine, Glu: glutamate, and DA: dopamine. Scale bar: 5 mm.

The ability of certain black yeasts, in particular of the family *Herpotrichiellaceae*, to assimilate cyclic aromatic hydrocarbons as a sole carbon source has been suggested to cause brain infections [6]. Figure 2a,c show representative experiments of quantitative analysis, which is presented in the Supplemental Material (Tables S1 and S2). We explored the assimilative ability of strain EXF-10123 by growing it on three different hydrocarbons: toluene, mineral oil, and n-hexadecane (Figure 2a,b). When growth of EXF10123 on solid media with or without exposure to toluene was compared, more abundant melanisation occurred under a toluene atmosphere. Colonies grew slower, but reached a similar size (5 +/− 1.5 mm) as on the control medium with added glucose. In liquid assimilation tests with added mineral oil, n-hexadecane, or glucose as a sole carbon source, EXF-10123 visually showed similar, floccular growth, yet with somewhat more pronounced growth on glucose.

We then investigated the ability of EXF-10123 to use common brain neurotransmitters, involved in fast synaptic transmission as a sole carbon source. EXF-10123 was inoculated on M9 solid minimal medium supplemented with either acetylcholine (ACh) or amino-acid based neurotransmitters (γ-aminobutyric acid (GABA), glycine (Gly), glutamate (Glu), or dopamine (DA)). Growth was monitored for four weeks (Figure 2c). EXF-10123 grew on all selected hydrocarbons and neurotransmitters.

The addition of neurotransmitters ACh and GABA promoted a higher growth rate compared with control (M9), while ACh stimulated dark pigmentation as well. ACh and Glu inhibited extension of visible hyphae, whereas hyphae were present after 14 days of incubation with GABA, Gly, and Glu. The colour of the colonies varied from light brown to dark brown or black when ACh and GABA were present. In general, ACh and GABA stimulated colony growth and melanisation, while Gly and Glu diminished growth by approximately 20%. A distinct colour change of the growth medium to dark blue and then to black took place only when DA was added as catecholamine neurotransmitter supplement, while the colonies themselves were lighter in colour and less dense when grown on DA as compared with other neurotransmitters.

### 3.3. Cytotoxicity of Metabolites Present in E. dermatitidis Extracts on SH-SY5Y Cells

**Figure 3:**
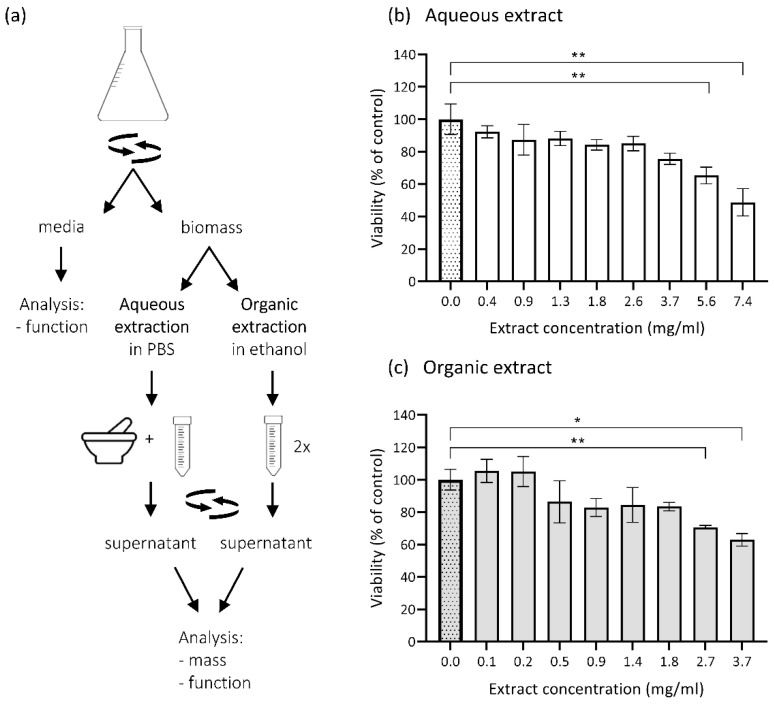
Cytotoxic effect of *E. dermatitidis* extracts on neuroblastoma cells SH-SY5Y. (**a**) Biomass extraction of four-day-old *E. dermatitidis* culture was performed according to the scheme. Cytotoxic effects of *E. dermatitidis* extracts were analysed on SH-SY5Y cells. Cells were cultured to 60%–70% confluence and treated with various concentrations of (**b**) aqueous or (**c**) DMSO-diluted ethanolic extracts. After 48 h, the neutral red uptake assay was performed and the cell viability was assayed as described in Section 2.10. Each bar represents mean ± SD of three biological repetitions. The first dotted bar represents the viability of the control cells (100%) in culture medium supplemented with (**b**) phosphate buffered saline (PBS) or (**c**) DMSO (*p < 0.05; **p < 0.01; Kruskal–Wallis statistics).

The possible indirect influence or cytotoxic effect of the conditioned fungal medium (without the fungus) and fungal metabolites on human neuroblastoma cell culture SH-SY5Y as a model for neuronal cells was checked. Fungal metabolites in aqueous and DMSO-dissolved ethanolic extracts were prepared from the biomass of EXF-10123 following the steps described in Figure 3a. SH-SY5Y cells were treated with different concentrations of extracts and with conditioned fungal medium. The viability of the exposed SH-SY5Y cells was assessed by neutral red uptake (NRU) viability assay. Addition of aqueous extracts at the lowest (0.4 mg dry weight/mL) and at the highest concentrations (7.4 mg dry weight/mL) decreased the viability by 8% and 51%, respectively (with statistical significance *p* = 0.0017; Kruskal–Wallis statistics) (Figure 3b). In comparison, the highest concentration (3.7 mg dry weight/mL) of organic extract lowered SH-SY5Y viability by 37% (*p* = 0.0017) (Figure 3c). The conditioned fungal medium had a neglectable cytotoxic effect on SH-SY5Y (Figure S1).

Exposure of the neuroblastoma cells SH-SY5Y to potential volatile organic compounds (VOC) from 1-, 2-, or 8-day-old EXF-10123 culture, or to the fresh fungal medium, did not significantly influence viability as compared with untreated control as tested with trypan blue staining after 48 h.

### 3.4. Isolation and Characterisation of E. dermatitidis EVs and Their Cytotoxic Effect on SH-SY5Y Cells

**Figure 4:**
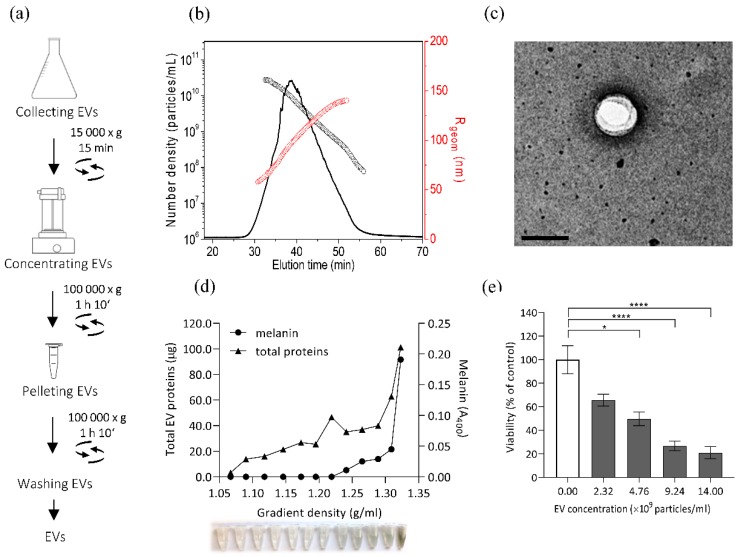
Extracellular vesicles (EVs) from *E. dermatitidis* show cytotoxic effects on SH-SY5Y cells. (**a**) EV isolation from 15 h old *E. dermatitidis* culture was performed according to the scheme. (**b**) *E. dermatitidis* EVs were characterized for morphology by asymmetrical-flow field-flow fractionation analysis coupled to a multidetection system (AF4-MALS). Normalized AF4-MALS fractogram (black line) of EVs for R_geom_ (red circles) and number density (black circles) is presented. (**c**) Transmission electron microscopy (TEM) micrograph of EV; scale bar: 200 nm. (**d**) *E. dermatitidis* EVs were subjected to velocity gradient centrifugation and the collected fractions were analysed for density, total EV proteins (µg), and melanin content (A_400_). (**e**) The cytotoxic effect of *E. dermatitidis* EVs on SH-SY5Y cells, as measured by the neutral red uptake viability assay. Data are presented as the mean ± SD of two independent experiments, where the white bar represents viability of control without added EVs (100%) (*p < 0.05; ****p < 0.0001; Kruskal–Wallis statistics).

Extracellular vesicles (EVs) released from *E. dermatitidis* culture could possibly have an effect on SH-SY5Y cells, as recent studies of cargo from fungal EVs revealed a complex protein composition with important roles in interspecies communication and pathogenicity [81,82]. To determine whether EXF-10123 releases vesicles, we optimized the EV isolation protocol based on our previous work with human cell cultures [83] and *S. cerevisiae* [84]. EVs were collected from 400 mL of EXF-10123 cultures and resuspended in 60 µl of DPBS, as shown in Figure 4a. The isolated particles had an average radius (*R*_geom_) of 90 nm and total concentration of 1.05 × 10^7^ particles per mL of media (Figure 4b), a typical EVs morphology (Figure 4c), and adequate purity. To further characterize these particles, they were separated on sucrose gradient (Figure 4d). Fractions with densities of 1.09–1.32 g/mL contained varying amounts of proteins, with the highest amounts in the densest fraction. We conclude that the isolated particles are EVs with typical morphology and buoyant density. Melanin was only present in a subset of fractions (densities 1.24–1.32 g/mL).

As *E. dermatitidis* is capable of causing brain infections, we analysed the effect of EVs from EXF-10123 on SH-SY5Y cells. SH-SY5Y viability decreased with increasing EV concentration, with the highest concentration of 1.4 × 10^10^ EVs/mL decreasing viability to 21% compared with control without added EVs (with statistical significance *p* < 0.0001).

### 3.5. Cytotoxic Effect on SH-SY5Y Cells is Significantly Lower with Non-Melanised EVs

**Figure 5:**
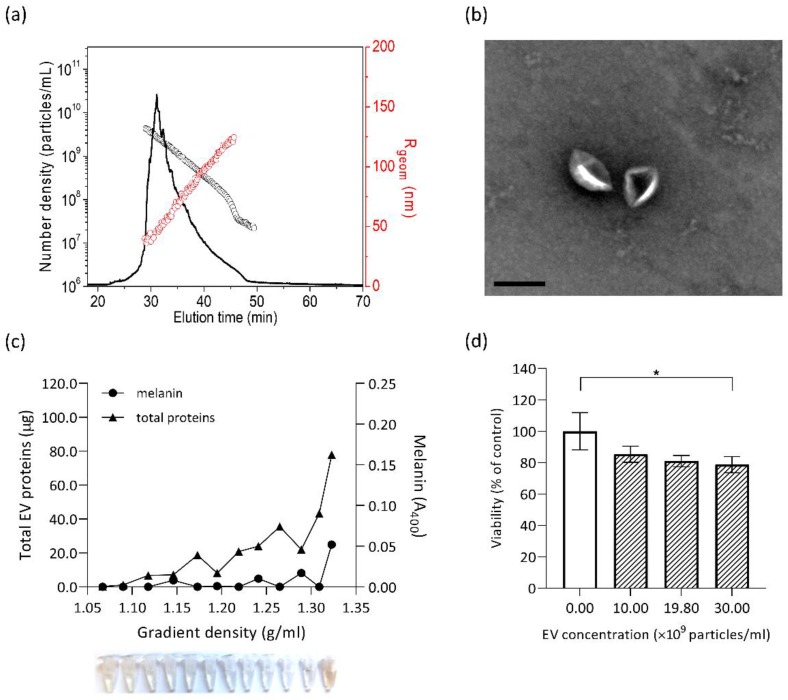
Melanin component of the *Exophiala dermatitidis* EVs is responsible for the SH-SY5Y cytotoxic effect. Morphological and molecular characterisation of *E. dermatitidis* EVs isolated from culture with added tricyclazole. (**a**) Normalized AF4-MALS fractogram (black line) of non-melanised EVs for R_geom_ (red circles) and number density (black circles). (**b**) TEM micrograph of non-melanised *E. dermatitidis* EVs; scale bar: 250 nm. (**c**) Velocity gradient centrifuged fractions of non-melanised *E. dermatitidis* EVs were collected and analysed for density, total EV proteins (µg), and melanin content (A_400_). (**d**) The cytotoxic effect of non-melanised *E. dermatitidis* EVs, added at the indicated final concentrations, on SH-SY5Y cells, as measured by the neutral red uptake viability assay. Data are presented as the mean ± SD of triplicate samples inside one experiment where the first, uncoloured bar represents viability of control without added EVs (100%) (*p < 0.05; Kruskal–Wallis statistics).

Melanin is known as an important virulence factor in black yeast-like fungi, particularly within *Chaetothyriales*. Here, we showed that a subpopulation of EXF-10123 EVs (1.24–1.32 g/mL density) was melanised. To determine the cytotoxic effect of melanin co-isolated with EVs (Figure 4) on neuroblastoma cells, we cultivated the fungus in a medium supplemented with the melanin-biosynthesis inhibitor tricyclazole prior to isolation of EVs. To isolate EVs from conditioned media, we followed the optimized protocol (Figure 4a) and characterized them with asymmetrical-flow field-flow fractionation analysis coupled to a multidetection system (AF4/UV-MALS) for particle size and concentration and transmission electron microscopy (TEM) for morphology. Additionally, we separated isolated EVs on sucrose density gradient and spectroscopically analysed collected fractions for protein and melanin content. Both with and without the presence of tricyclazole, EXF-10123 released EVs in high concentrations (1.5 × 10^11^ EVs/mL; Figure 5a) and with a typical cup-shaped morphology under TEM (Figure 5b) and typical buoyant density, as shown by the presence of proteins in denser gradient fractions (1.22–1.32 g/mL, Figure 5c). However, EVs released in the presence of tricyclazole were smaller than in the absence of the inhibitor (the average radius *R*_geom_ as determined by AF4/UV-MALS was 75 nm vs. 90 nm; Figure 5a and Figure 4b). Furthermore, melanin was absent in most gradient fractions (Figure 5c), suggesting that EXF-10123 releases EVs without melanin in the presence of the melanin-inhibitor tricyclazole.

To compare the function of non-melanised EXF-10123 EVs with those containing melanin, we performed a neutral red uptake viability assay, as described above. After exposure to SH-SY5Y cells, such non-melanised EVs, even at the highest concentration of 3.0 × 10^10^ EVs/mL, showed only minor cytotoxic effect on SH-SY5Y cells, with 79% viability compared with control (statistical significance *p* = 0.0347; Figure 5d). We conclude that the melanin component of EXF-10123 EVs is important for the cytotoxic effect on SH-SY5Y cells (see the comparison on survival diagram, Figure S4).

### 3.6. Direct Interaction of E. dermatitidis with SH-SY5Y Cells Shows Fungal Internalisation Followed by Neuroblastoma Cell Death

**Figure 6:**
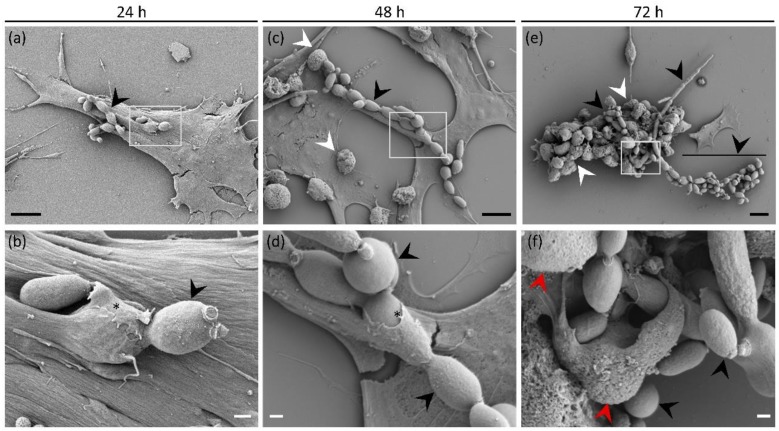
Internalization of *Exophiala dermatitidis* into neuroblastoma cells SH-SY5Y during three day co-cultivation observed by a scanning electron microscope (SEM). The columns represent 24 h, 48 h, and 72 h of co-cultivation of the fungus and SH-SY5Y cells. Images in the lower row depict enlarged insets of images above them. (**a**) Neuroblastoma cells attached to the surface after 24 h co-cultivation, with partially internalized pseudohyphae (black arrowheads) on their surface. (**b**,**d**) Note pseudopodia extending over fungal cell (asterisk). (**c**,**d**) Continuous growth of pseudohyphae (black arrowheads), their internalization, and (**c**) gradual degradation of neuroblastoma cells by formation of cell blebs (white arrowheads) on the cell surface, after 48 h of co-cultivation. (**e**,**f**) Degradation of neuroblastoma cells by extensive fungal growth seen as blebbing (white arrowheads) and (**f**) degradation of the cell membrane (red arrowheads) after 72 h of co-cultivation. Black and white scale bars represent 10 μm and 1 μm, respectively.

Different virulence factors allow fungi to bypass the immune system, enter the body of a healthy individual through various pathways, and cause deep mycoses such as CNS infections. To investigate the potential for CNS invasion of *E. dermatitidis*, we inoculated SH-SY5Y cells with EXF-10123 yeast cells and observed the interaction at 24, 48, 72, and 96 h by scanning electron microscopy (SEM) and confocal laser scanning microscopy. Twenty-four hours after inoculation, the yeast cells prolonged into pseudohyphae and appeared firmly attached to the surface of SH-SY5Y cells (Figure 6a). Observation at a higher magnification revealed gradual internalization of yeast-like cells by extension of neuroblastomal pseudopodia over them (Figure 6d), whereas the general morphology of SH-SY5Y cells appeared normal and comparable to the control group grown without the fungus (Figure S2). Further internalization of yeast-like cells (Figure 6e) accompanied by continuous growth of pseudohyphae (Figure 6b) were observed after 48 h of incubation. The first signs of SH-SY5Y cell degradation appeared as blebbing of the plasma membrane (Figure 6e). After 72 h of co-cultivation (Figure 6c), the growing fungus degraded SH-SY5Y cells that subsequently became hardly recognizable, comprising predominately of cell blebs with damaged cell membranes (Figure 6f). Fungal overgrowth, as well as degradation and peeling of SH-SY5Y cells, also prevented any sample preparation beyond 72 h (Figure S3).

**Figure 7:**
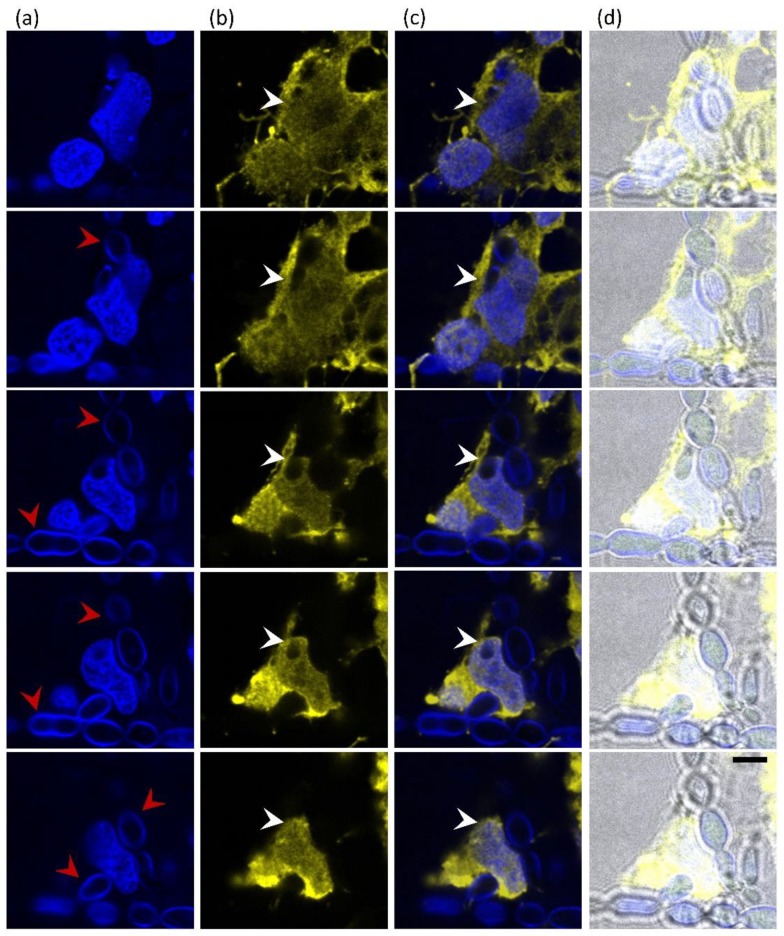
Internalization of *Exophiala dermatitidis* into neuroblastoma cells SH-SY5Y after 48 h incubation. Five confocal images, imaged as Z-stack (top to bottom row), show the interaction of SH-SY5Y neuroblastoma cells with *E. dermatitidis*. (**a**) Fungi were detected with Calcofluor White (CFW) and SH-SY5Y nuclei with DAPI, both in blue. Red arrows point to pseudohyphae. (**b**) SH-SY5Y cytoplasm was stained in yellow using anti-GAPDH antibodies and secondary antibodies conjugated to Alexa 555. Penetrating fungal cells in the cytoplasm of neuroblastoma cells are clearly visible in the sequential cell sections (white arrows). (**c**) Merged images from images (**a**) and (**b**), (**d**) merged images including bright field (BF). Scale bar: 5 μm.

Interactions of EXF-10123 and SH-SY5Y cells were also observed by confocal laser scanning microscope, in order to determine specific components of the interaction and the potential penetration into SH-SY5Y cells. Black yeasts are extremely hard to mark with fluorochromes, because of their thick and melanised cell walls. Therefore, fungal cells were marked with Calcofluor white (CFW), a non-specific fluorochrome that binds to cellulose and chitin in cell walls. To distinguish between SH-SY5Y cells and fungi and to display internalization, specific primary mouse monoclonal anti-GAPDH antibody and secondary anti-mouse Alexa Fluor^®^ 555 IgG H&L were used to mark SH-SY5Y cytoplasm (Figure 7). After two days of incubation, interactions of EXF-10123 and SH-SY5Y cells were observed as EXF-10123 pseudohyphae were invading SH-SY5Y cells (Figure 7b,c; third row, white arrows). Where the fungus was engulfed by neuroblastoma cells, direct contact between both organisms could be determined (Figure 7b,c; Z-stack rows 1–5). Z-stack images of fungal pseudohyphae internalized into SH-SY5Y cells showed that the fungus indeed penetrated those cells (not just establishing contact points with the outer membrane; Figure 7b) and promoted cell death, as imaged by SEM (Figure 6).

## 4. Discussion

In this study, we investigated the main features responsible for the pathogenicity of *E. dermatitidis*. Its neurocytotoxic effect was determined on the human neuroblastoma cell line SH-SY5Y. These cells represent suitable in vitro models for the determination of cytotoxicity [85,86] and for neurological studies, including of Alzheimer’s disease [85].

*Exophiala dermatitidis* is rarely isolated from nature, but frequently and globally from domestic environments and particularly house appliances containing plastic and rubber-like materials [35,36,37,38,39,87]. Thus, it represents an important model organism for studying interactions between a “domesticated” fungus and the neuroblastoma cells SH-SY5Y owing to its virulence factors, that is, tolerance to human body temperature (37 °C), polymorphism, melanisation, oligotrophicity, ability to assimilate aromatic compounds, and related neurotropism [3,5,6,8,48,53,88,89].

Here, we have shown that several human neurotransmitters are excellent substrates for *E. dermatitidis* EXF-10123, as it was possible to assimilate volatile toluene and other aromatic or phenolic hydrocarbons as sole carbon sources. CNS is a rich source of several monoaromatic catecholamine neurotransmitters (e.g., dopamine, serotonin, nor-epinephrine) and aliphatic amino alcohols that are potential substrates for neurotropic fungi [50,51]. CNS also harbours large amounts of ACh and amino acid-based neurotransmitters like Glu, GABA, and Gly, which are involved in synaptic transmission and could represent alternative substrates for invading fungi. It is tempting to speculate that fungi colonizing the brain could contribute to decreased ACh levels and to the gradual loss of cholinergic transmission, one of the common deficiencies in Alzheimer’s.

Exposure of neuroblastoma cells to both aqueous and organic extracts of EXF 10123 significantly diminished their viability. This suggests that the fungus harbours cytotoxic compounds with different polarities, and that it does not exert its cytotoxicity via volatile organic compounds.

Extracellular vesicles (EVs) may expose neuroblastoma cells to *E. dermatitidis* metabolites. EVs are known to affect pathogenesis and cell communication through the delivery of enzymes, toxins, communication signals, and antigens recognized by the innate and adaptive immune systems [68,82,90,91,92]. EVs from fungi pass through the fungal cell wall by mechanisms that are not yet understood [70,71,93]. The composition of their cargo is strongly connected to their virulence [81,82], indicating the importance of EVs in fungal pathogenesis and as a potential target for protection against fungal infections.

Further, melanin is strongly connected to virulence and is known to activate the host’s immune system [94]. Melanin is not only bound to the cell wall, but can also be transported out of the cell embedded in EVs as melanin granules, as has been shown for the opportunistic pathogenic fungus *Cryptococcus neoformans* [95]. The attachment of melanin to the cryptococcal cell wall and the detection of 200 nm spherical structures were also reported by Camacho et al. [96]. The other melanin study by Upadhyay et al. [97] showed the trafficking of melanin enzymes to endosomes or multivesicular bodies (MVBs), which are a possible biosynthetic machinery of EVs. EXF-10123 secretes EVs both with and without melanin included in their cargo. EVs containing melanin reduced the viability of neuroblastoma cells significantly more than those without. The fact that the cytotoxic effect of *E. dermatitidis* EVs is strongly dependent on their cargo of melanin underlines the importance of melanin as a virulence factor. Note that EVs from non-melanised *Saccharomyces cerevisiae* (BY4741) did not have any cytotoxic effect on SH-SY5Y cells (unpublished).

Fungal brain infections (mostly by in vivo imaging evidence) are increasingly being reported [57,98,99], including reports related to diagnostics [100,101]. Additionally, in vivo intravital microscopy (IVM) produced some evidence of fungal invasion into brain vasculature [102]. Despite this, traversal pathways of fungal species or metabolites are mostly unknown, although some aspects of crossing the blood–brain barrier [103,104] and enhancement of brain infection via cell-derived vesicles [69] are well established.

We also exposed neuroblastoma cells SH-SY5Y to EXF-10123 by co-cultivation. Microscopy indicated that pseudo-hyphae of EXF-10123 penetrated human neuroblastoma cells, thereby presumably causing their death in three days. Initially, EXF-10123 grew as a budding yeast. After 24 h, it differentiated into pseudohyphae that established contact with SH-SY5Y cells. Surprisingly, SH-SY5Y cells reacted by the formation of pseudopodia that grew over the fungal hyphae and apparently embedded them. After two days, the neuroblastoma cells started to show signs of decay and degradation similar to apoptosis, characterized by blebbing of the plasma membrane [105]. Confocal micrograms also revealed an increasing number of nanotubes (Tunnelling Nanotubes, TNT) consisting of thin, extended membrane protrusions that connect cells and transfer various cellular components [106,107] and extracellular vesicles [108,109]. After three days, the neuroblastoma cells were completely degraded, resulting in their detachment on the fourth day. Confocal laser scanning microscopy supplemented these observations, showing penetration of hyphae into neuroblastoma cells and even penetration into their nuclei. Our results show both cytotoxicity to the SH-SY5Y cells after exposure to EXF-10123 metabolites as well as progressive cell death upon direct exposure of neuroblastoma cells to this fungus. To the best of our knowledge, invasion of neuroblastoma cells with any fungi has not yet been reported.

## 5. Conclusions

In conclusion, we report several novel results and observations related to the extremophilic black yeast *Exophiala dermatitidis* strain EXF-10123 and its possible relation to human neurodegenerative diseases. We showed that both organic and aqueous extracts of this fungus significantly reduced the viability of human neuroblastoma cells, as did secreted extracellular vesicles. Vesicles containing melanin reduced the viability of neuroblastoma cells significantly more than those without. Direct cultivation of EXF-10123 and neuroblastoma cells showed penetration of fungal hyphae into human neuroblastoma cells, followed by their death in three days. These observations may reflect a new pathway for the traverse of fungi or fungal metabolites into the brain, hence crossing the blood–brain barrier. The above lends credibility to the hypothesis that some extremophilic fungi, such as *E. dermatitidis*, may be a direct or indirect cause of some neurodegenerative diseases such as Alzheimer’s.

## Supplementary Materials

The following are available online at https://www.mdpi.com/2073-4409/9/4/963/s1, Figure S1: Cytotoxic effect of *Exophiala dermatitidis* conditioned medium (CM) on cells SH-SY5Y; mean ± SD of three biological repetitions. Figure S2: Micrographs of uninfected cells SH-SY5Y. Figure S3: Micrographs of internalization of *Exophiala dermatitidis* into SH-SY5Y cells. Figure S4: Survival diagram of cytotoxic effect of melanised *Exophiala dermatitidis* EVs compared with non-melanised EVs. Table S1: Semi-quantification of the intensity of plate images of *Exophiala dermatitidis* (EXF-10123). Table S2: Live/dead staining of fungus *Exophiala dermatitidis* grown in medium without (ED w/o) or with (ED w) added inhibitor tricyclazole.
