# Supplementary material for: The Neurotropic Black Yeast Exophiala dermatitidis Induces Neurocytotoxicity in Neuroblastoma Cells and Progressive Cell Death
Source: Cells. 2020 Apr 14;9(4):963. doi: 10.3390/cells9040963 (PMC7226985; doi:10.3390/cells9040963)
Supplement: Supplementary file 1 [file cells-09-00963-s001.pdf]

SUPPLEMENTAL TABLE 1 Semi-quantification of the intensity of plate images of *Exophiala dermatitidis* (EXF-10123). Growth was tested on (A) selected cyclic aromatic hydrocarbons and (B) neurotransmitters as sole carbon source. Images were analysed by Image J; results are given as mean grey value (MGV) in pixels by calculation: grey = (red + green + blue) = 3. Higher MGVs represent slower growth and lower melanisation, while lower MGVs represent reverse effect.

A Toluene assimilation on solid medium

Mean grey value (pixels)					
YNB + glucose		YNB		agar	
toluene	73.755	toluene	126.427	toluene	107.41
air	98.907	air	137.59	air	118.222

B Neurotransmitters assimilation (Ach: Acetylcholine, GABA: gamma-Aminobutyric acid, Gly: Glycine, Glu: Glutamate, DA: Dopamine), monitored for four weeks

Mean grey value (pixels)									
	25 °C					25 °C		37°C	
	M9	ACh	GABA	Gly	Glu	M9	DA	M9	DA
7 days	122.267	91.312	115.128	135.748	137.939	68.651	56.524	41.532	61.828
14 days	97.736	46.331	91.421	124.923	115.61	37.403	83.579	21.246	41.312
21 days	71.364	36.424	61.436	105.341	108.612	51.011	62.2	20.065	47.422
28 days	67.086	43.216	65.318	107.466	94.484	45.566	66.575	23.04	38.324

SUPPLEMENTAL TABLE 2 Live/dead staining of fungus *Exophiala dermatitidis* grown in medium without (ED w/o) or with (ED w) added inhibitor tricyclazole. The procedure was performed according to Nelson et al. (2000); viability is given as a percentage (%)

Sample	Viability percent (%)
ED w/o	89.6
ED w	90.4

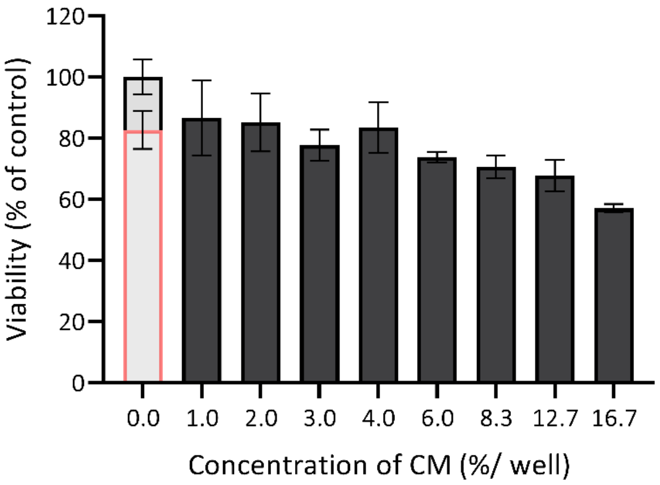

SUPPLEMENTAL FIGURE 1 Cytotoxic effect of *Exophiala dermatitidis* conditioned medium (CM) on cells SH-SY5Y; mean ± SD of three biological repetitions. The first overlapping red and black bars represent the viability of control with 8.3% and 4% of ME medium, respectively

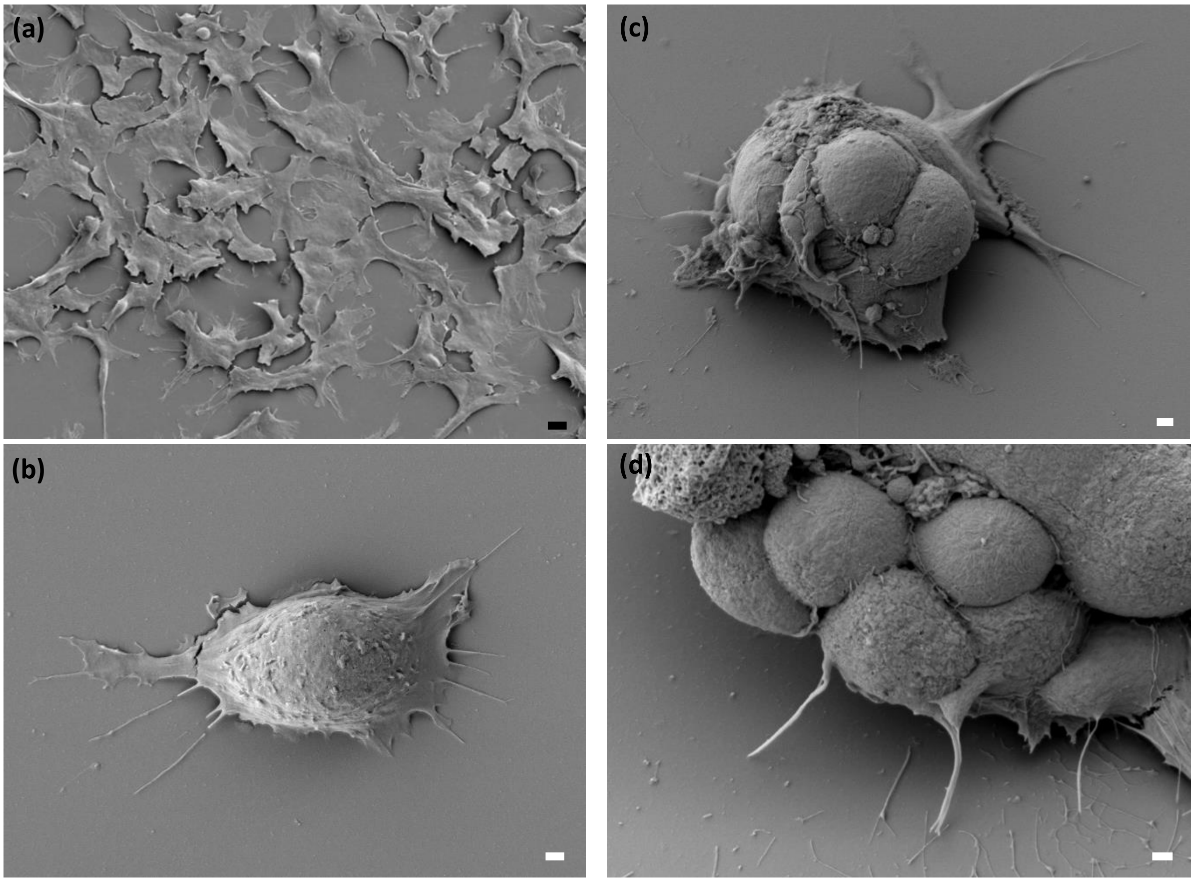

SUPPLEMENTAL FIGURE 2 Micrographs of uninfected cells SH-SY5Y; (a) general morphology of normal confluent cells with (b) magnified details and (c,d) natural cell process of membrane blebbing and signs of cell decay; scale bars: 10 µm (black) and 1 µm (white)

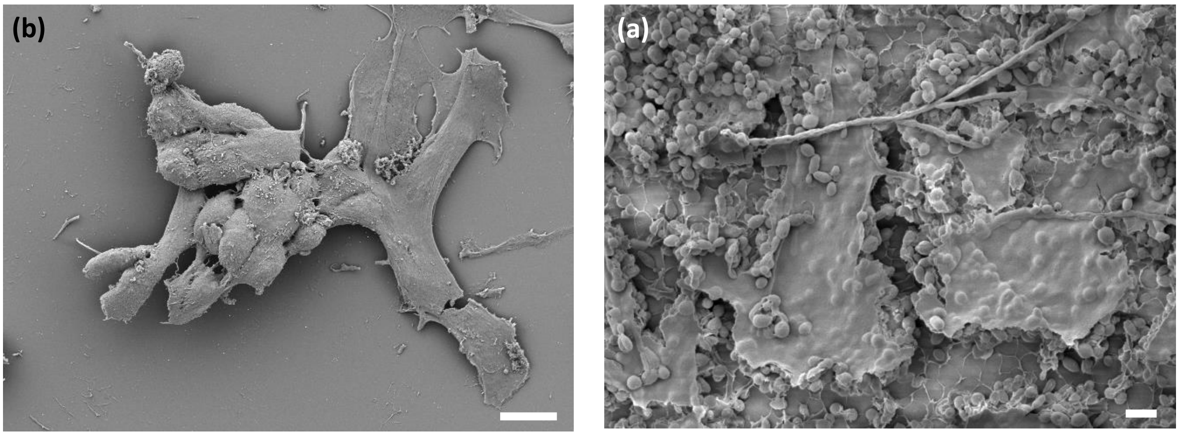

SUPPLEMENTAL FIGURE 3 Micrographs of Internalization of *Exophiala dermatitidis* into SH-SY5Y cells; (a) after 4-day and (b) 5-day co-cultivation. Both represent two different biological repetitions with two different SH-SY5Y densities; scale bar: 10 µm

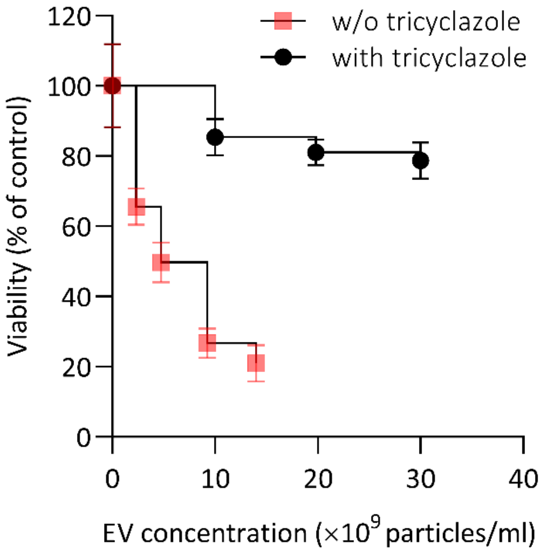

SUPPLEMENTAL FIGURE 4 Survival diagram of cytotoxic effect of melanised *Exophiala dermatitidis* EVs (w/o tricyclazole; red squares) compared to non-melanised EVs (with tricyclazole; black dots) added at the final concentrations on SH-SY5Y cells, measured by NRU viability assay. Scatter plots represent mean ± SD of triplicate samples
